# Exploring tRNA gene cluster in archaea

**DOI:** 10.1590/0074-02760180348

**Published:** 2019-01-07

**Authors:** Sergio Mascarenhas Morgado, Ana Carolina Paulo Vicente

**Affiliations:** Fundação Oswaldo Cruz-Fiocruz, Instituto Oswaldo Cruz, Laboratório de Genética Molecular de Microrganismos, Rio de Janeiro, RJ, Brasil

**Keywords:** archaea, tRNA gene cluster, plasmid, haloarchaea, virus, Euryarchaeota

## Abstract

**BACKGROUND:**

Shared traits between prokaryotes and eukaryotes are helpful in the understanding of the tree of life evolution. In bacteria and eukaryotes, it has been shown a particular organisation of tRNA genes as clusters, but this trait has not been explored in the archaea domain.

**OBJECTIVE:**

Explore the occurrence of tRNA gene clusters in archaea.

**METHODS:**

*In-silico* analyses of complete and draft archaeal genomes based on tRNA gene isotype and synteny, tRNA gene cluster content and mobilome elements.

**FINDINGS:**

We demonstrated the prevalence of tRNA gene clusters in archaea. tRNA gene clusters, composed of archaeal-type tRNAs, were identified in two *Archaea* class, *Halobacteria* and *Methanobacteria* from Euryarchaeota supergroup. Genomic analyses also revealed evidence of the association between tRNA gene clusters to mobile genetic elements and intra-domain horizontal gene transfer.

**MAIN CONCLUSIONS:**

tRNA gene cluster occurs in the three domains of life, suggesting a role of this type of tRNA gene organisation in the biology of the living organisms.

The three domains of life, *Archaea*, *Bacteria* and *Eukarya* are characterised by unique features, even though they share a set of basic characteristics. *Archaea* and *Bacteria* share ribosomal genes, transcriptional regulators and overall genome structure, while archaea and eukaryotes are more related about the information transfer system, including replication, transcription and translation processes.^1^ ^,^ ^2^ Transfer RNAs (tRNAs) have a major role in the translation machinery and, therefore, belong to the core system of the three domains of life. This molecule is associated with a huge diversity of gene organisation, including canonical and disrupted genes. The latter gene species was revealed to be prevalent among archaea genomes.^3^ The tRNA genes are found dispersed in the genomes but occasionally, as in mitogenomes, they are organised in clusters containing two to five tRNA genes.^4^ Besides mitogenomes, tRNA gene clusters have been shown to be abundant in *Eukarya*.^5^ In prokaryotes, tRNA gene clusters were characterised in some bacteria, being prevalent in *Firmicutes* phylum and *Mycobacterium* genus,^6^ ^,^ ^7^ while in archaea, the presence of one tRNA gene cluster was only identified in the *Methanobrevibacter* M1 strain genome.^6^ In addition, tRNA gene clusters have also been particularly observed in mycobacteriophages.^7^ ^,^ ^8^ In this case, these tRNA genes could be useful in the late lytic growth overcoming the degradation and/or differences in the codon bias of the host tRNAs.^8^ The prevalence of tRNA gene clusters in bacteria, eukaryotes and bacteriophages raise a possible role of this type of gene organisation in the biology of these organisms. The presence of tRNA genes organised in a cluster, some encoding for all the tRNA isotypes, as well as unusual tRNA isotypes (e.g., selenocysteine, pyrrolysine), would provide to the organisms a set of genes basic to protein synthesis in a one-step acquisition way, what may positively impact their fitness. Identifying tRNA gene clusters in the three domains of life is likely to reveal their evolutionary history as well any current function. Therefore, we hypothesised the prevalence of tRNA gene cluster in archaea. In order to test our hypothesis, we analysed 2481 complete and draft archaea genomes overrepresented by Euryarchaeota supergroup.

## MATERIALS AND METHODS

*Genomes analysed* - A total of 2481 complete and draft archaeal genomes (available in May 2018) were retrieved from National Center for Biotechnology Information (NCBI) FTP site (ftp://ftp.ncbi.nlm.nih.gov/genomes/genbank/archaea/).

*tRNA gene prediction and tRNA gene cluster identification* - The tRNA gene prediction of the data set was primarily performed by tRNAscan-SE 2.0^9^ using the archaeal model, and inconsistent predictions were verified using ARAGORN v1.2.38.^10^ Here we surveyed tRNA gene clusters with a minimum of 10 tRNA genes and used the approach described in a previous study to identify them.^7^ tRNA genes were considered clustered if presented a tRNA gene density ≥ 2 tRNA/kb.^5^

*Genetic analysis* - The genetic relationships of the genomes harboring tRNA gene clusters (Euryarchaeota supergroup) were assayed performing a core genome Multilocus Sequence Analysis (cgMLSA). The orthologous genes were retrieved using GET_HOMOLOGUES v3.0.5^11^ considering a coverage of ≥ 70% and identity ≥ 40%. The 66 orthologous genes were concatenated (yielding ~75 kb length) and submitted to Maximum-likelihood analysis using PhyML v3.1.^12^ Genetic relationships of the tRNA genes from the tRNA gene clusters were also assessed by concatenation of their nucleotide sequence (~3 kb length), and subsequent Maximum-likelihood analysis. The alignments were performed by MAFFT v7.271,^13^ and the tree figures generated by iTOL.^14^

*Sequence annotation, plasmid, and viral identification* - The sequence of tRNA gene cluster regions and their flanking regions (~10 kb) were annotated using Prokka v1.12.^15^ A bipartite network of gene content was build using AcCNET v1.2^16^ and visualised in Cytoscape v3.6.0.^17^ The topology of the contigs harboring tRNA gene clusters was assayed as described by Jørgensen et al.^18^. These contigs were also submitted to blastn and virSorter^19^ analysis for plasmid and viral/proviral identification, respectively.

## RESULTS

*Detection and distribution of tRNA gene clusters among archaea genomes* - In order to identify the presence of tRNA gene clusters in archaea genomes, we used the methodology described in a previous study.^7^ The analysis identified tRNA gene clusters in 29/2481 archaea genomes from GenBank (Table). The number of tRNA genes in the arrays ranged from 10 to 29, including five clusters with 20 or more tRNA genes. The tRNA clusters encompassed regions from ~1.1 to ~7 kb, representing a density from ~2.14 to ~9.17 tRNA gene/kb (Table). The number of tRNA genes present in these clusters corresponds to ~ 17 to 46% of the total number of tRNA genes in these archaea genomes. Two genomes, *Methanobrevibacter* sp. RUG344 and *Methanobrevibacter* sp. RUG833 were the only ones harboring two tRNA gene clusters (Table).

**Fig. 1:**
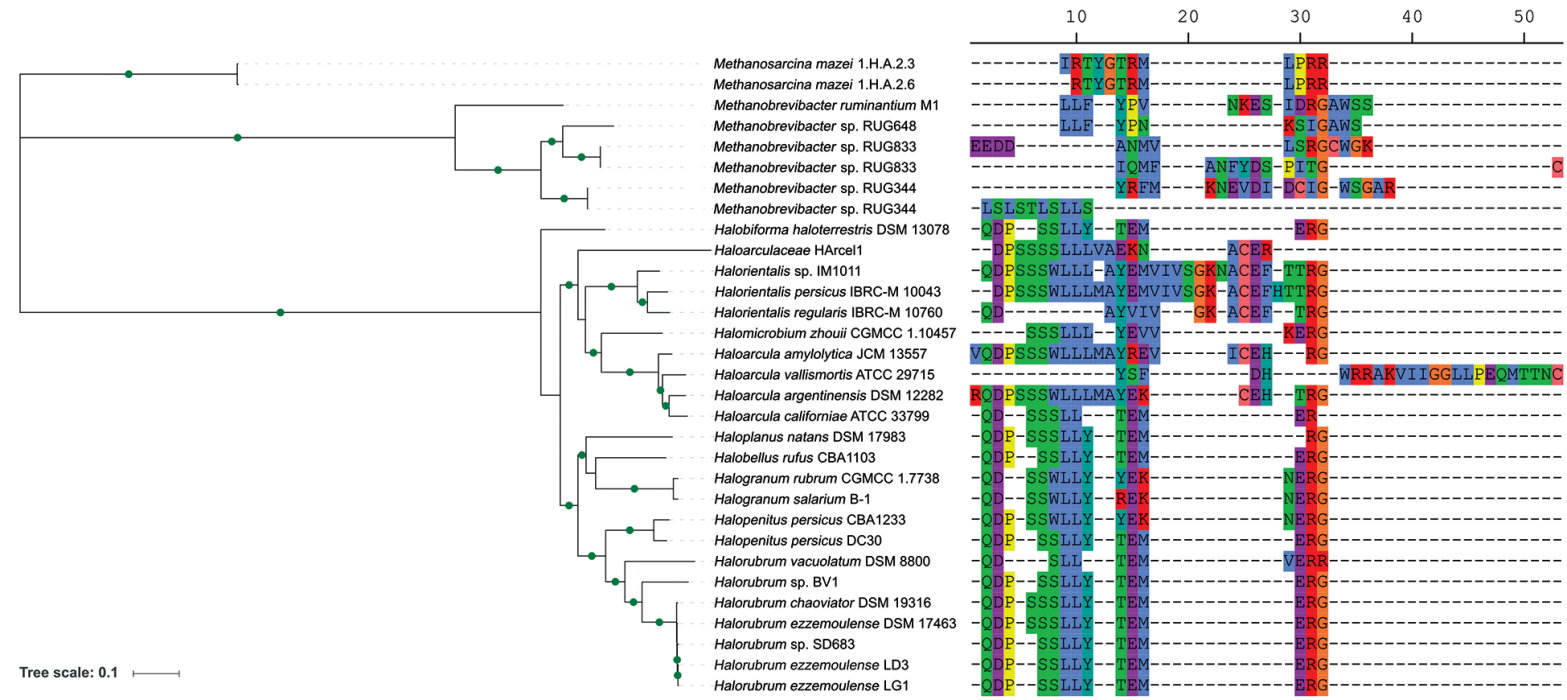
maximum likelihood tree based on 66 concatenated orthologous genes. In the right side, the tRNA isotype organisation (using the single-letter amino acid code) of the tRNA gene clusters from each genome is related to the tree branches. The gaps (- symbol) may not represent the actual distance between two adjacent tRNA genes, but the distance from the reference tRNA gene cluster. The green circles in the branches indicate bootstrap values ≥ 70.

**Fig. 2:**
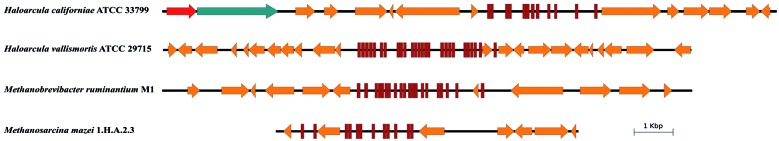
linear representation of the tRNA gene cluster genomic regions of some representative genomes. The orange arrows and red bars represent CDS and tRNA genes, respectively. In *Haloarcula californiae* representation the red and blue arrows indicate the genes encoding for Relaxase and conjugative transfer protein TraD, respectively.

The 29 genomes harboring the tRNA gene clusters correspond to 18 species and seven spp. belonging to 11 archaea genera, all from Euryarchaeota supergroup, including *Haloarcula*, *Halobellus*, *Halobiforma*, *Halogranum*, *Halomicrobium*, *Halopenitus*, *Haloplanus*, *Halorientalis*, *Halorubrum*, *Methanobrevibacter*, and *Methanosarcina*. The latter two genera are from methanogenic archaea, while the others, halophilic. Analysing the sequences of the tRNA genes in the clusters, we observed that all but *Methanosarcina* are archaeal-type tRNAs. The phylogenetic analysis revealed that all genomes from *Halorubrum* genus but *Halorubrum vacuolatum* DSM 8800 and *Halorubrum* sp. BV1, belong to a single lineage as well as the two genomes from *Methanosarcina mazei* (Fig. 1). Strains harboring tRNA gene clusters with a same tRNA amino acid isotype pattern were isolated from different geographic locations, even considering species from the same lineage (Table). Despite the evolutionary relationship among some strains, their tRNA isotype organisation and genomic context differ substantially (Figs 1-2). Interestingly, 23/29 and 6/29 tRNA gene clusters were identified among 239 halophilic and 618 methanogenic archaea genomes, respectively, revealing a bias to the prevalence of tRNA gene clusters in halophilic archaea. On the other hand, tRNA gene clusters were not identified in any other archaea supergroup.

*tRNA gene cluster organisation and composition* - Overall, the halophilic archaea share tRNA gene clusters presenting similar tRNA amino acid isotype composition and synteny. The exception is the *Haloarcula vallismortis* ATCC 29715 which has a distinct tRNA amino acid isotype composition (Fig. 1). In contrast, the methanogenic archaea harboring tRNA gene clusters present a quite distinct tRNA isotype composition and synteny. This scenario is also observed when considering the nucleotide sequences of the tRNA genes from the clusters [Supplementary data (Fig. 1)]. The most represented isotypes in the tRNA gene clusters were tRNA-Leu and tRNA-Ser, with up to four gene copies per cluster, while the less frequent being tRNA-His and tRNA-Phe, absent in 25 and 21 tRNA gene clusters, respectively [Supplementary data (Table I)]. Only *H. vallismortis* ATCC 29715 tRNA gene cluster presented all universal 20 isotypes, while *Halorientalis* sp. IM1011 cluster only has tRNA-His absent. In 26/29 archaea genomes, the tRNA-Tyr and tRNA-Glu from tRNA gene clusters represent a significant mean increment (~45%) to the strains. In general, all tRNA isotypes present in the tRNA gene clusters are redundant with the tRNA isotype repertoire from archaea genomes, the exception is tRNA-Gly in *Methanobrevibacter* sp. RUG833, which is only present in its tRNA gene cluster [Supplementary data (Table I)]. However, we observed that for 9/29 genomes there was an increase of isoacceptors provided by some tRNA gene clusters, which increased from one to eight species, depending on the strain [Supplementary data (Table II)]. Almost all clusters presented tRNA genes annotated as pseudogenes. In *Methanobrevibacter* sp. RUG833, which harbors two tRNA gene clusters summing 31 tRNA genes, 17 tRNA genes were annotated as pseudogenes (Table). This was also observed among the *Halorientalis* genomes, which also showed some tRNA genes with introns.

*Gene content of archaea tRNA gene cluster* - The number of protein-coding genes accompanying the tRNA gene clusters is variable, most of them encoding hypothetical proteins. A gene encoding for a TROVE domain-containing protein is presented in 20/29 clusters [Supplementary data (Fig. 2)]. The majority of the genes shared by the clusters belong to the halophilic archaea (except for *H. vallismortis* and *Halogranum* genomes), therefore this group represents the basis of the tRNA gene content network in archaea [Supplementary data (Fig. 3)].

Several genes associated with DNA transfer mechanisms were identified in the genomic context of the tRNA gene clusters, including transposases, integrases, HNH endonucleases, relaxase, toxin-antitoxin systems and secretion systems related genes [Supplementary data (Table III)]. Besides these, there were also resistance genes to antibiotics/metals (teicoplanin, bleomycin, chloramphenicol, tetracycline, arsenic). Interestingly, *Halogranum salarium* and *Halogranum rubrum* genomes presented a gene encoding for a conjugal transfer protein (HerA). In the methanogenic archaea genomes, it was identified a gene encoding for a putative phage holin family protein (*Methanobrevibacter ruminantium* M1), and in *M. mazei* there were protein-coding genes with high similarity to bacteria, bacteriophages, and plasmids from *Proteobacteria* phylum [Supplementary data (Table IV)], including chloramphenicol O-acetyltransferase gene sequences [Supplementary data (Fig. 4)].

**TABLE:** Archaeal genomes harboring tRNA gene clusters

Genome	Genome (Mb)	# tRNAs (genome)	# tRNAs (cluster)	Length (bp)	BEGIN (bp)	END (bp)	Density (tRNA/kb)	Archaeal type	Isolation location	Access number	# pseudogenes	# introns
*Haloarcula amylolytica* JCM 13557	4.3	70	23	6311	21449	27759	3.64	Halophilic	China	AOLW01000050.1	2	-
*Haloarcula argentinensis* DSM 12282	4.2	71	22	6998	216765	223762	3.14	Halophilic	Argentina	AOLX01000027.1	2	-
*Haloarcula californiae* ATCC 33799	4.4	60	12	2825	9001	11825	4.24	Halophilic	-	AOLS01000072.1	2	-
*Haloarcula vallismortis* ATCC 29715	4.2	72	25	3569	254165	257733	7.00	Halophilic	USA	AOLQ01000004.1	3	1
*Haloarculaceae* archeaon HArcel1	2.7	66	18	5757	2632007	2637763	3.12	Halophilic	Russia	CP028858.1	1	-
*Halobellus rufus* CBA1103	3.8	60	14	4602	6479	11080	3.04	Halophilic	-	BBJO01000043.1	1	-
*Halobiforma haloterrestris* DSM 13078	4.4	61	14	5924	13582	19505	2.36	Halophilic	-	FOKW01000014.1	1	-
*Halogranum rubrum* CGMCC 1.7738	4.5	70	15	3838	160615	164452	3.90	Halophilic	-	FOTC01000006.1	1	-
*Halogranum salarium* B-1	4.5	70	15	3838	40762	44599	3.90	Halophilic	South Korea	ALJD01000014.1	3	-
*Halomicrobium zhouii* CGMCC 1.10457	4.2	62	14	5662	253014	258675	2.47	Halophilic	-	FOZK01000002.1	-	-
*Halopenitus persicus* CBA1233	2.9	65	16	3934	1151067	1155000	4.06	Halophilic	South Korea	AP017558.1	2	-
*Halopenitus persicus* DC30	3.4	61	14	4601	55752	60352	3.04	Halophilic	-	FNPC01000012.1	1	-
*Haloplanus natans* DSM 17983	3.8	66	14	6473	401627	408099	2.16	Halophilic	Israel	ATYM01000003.1	3	-
*Halorientalis persicus* IBRC-M 10043	5	81	29	5739	15276	21014	5.05	Halophilic	Iran	FOCX01000031.1	6	2
*Halorientalis regularis* IBRC-M 10760	4	66	16	6676	110496	117171	2.39	Halophilic	-	FNBK01000012.1	2	2
*Halorientalis* sp. IM1011	3.8	79	29	5677	2239040	2244716	5.10	Halophilic	China	CP019067.1	5	1
*Halorubrum chaoviator* DSM 19316	3.6	62	15	4553	11178	15730	3.29	Halophilic	Mexico	FZNK01000013.1	3	-
*Halorubrum ezzemoulense* DSM 17463	3.6	63	15	4606	2376	6981	3.25	Halophilic	-	NEDJ01000026.1	2	-
*Halorubrum ezzemoulense* LD3	3.7	60	14	4552	12826	17377	3.07	Halophilic	Iran	NHOW01000026.1	1	-
*Halorubrum ezzemoulense* LG1	4.6	72	14	4554	12827	17380	3.07	Halophilic	Iran	NHOV01000832.1	1	-
*Halorubrum* sp. BV1	2.7	63	14	4541	180660	185200	3.08	Halophilic	-	JQKV01000005.1	2	-
*Halorubrum* sp. SD683	3.1	62	14	4607	33841	38447	3.03	Halophilic	Namibia	NEWJ01000007.1	1	-
*Halorubrum vacuolatum* DSM 8800	3.4	61	12	3781	10871	14651	3.17	Halophilic	Kenya	FZNQ01000022.1	1	-
*Methanobrevibacter ruminantium* M1	3	57	18	3608	360196	363803	4.98	Methanogenic	-	CP001719.1	2	-
*Methanobrevibacter* sp. RUG344	2.3	63	19	3477	32563	36039	5.46	Methanogenic	Scotland	ONJC01000013.1	4	-
			10	1115	27774	28888	8.96			ONJC01000014.1	2	-
*Methanobrevibacter* sp. RUG648	2.3	40	13	2348	6705	9052	5.53	Methanogenic	Scotland	ONUN01000043.1	4	-
*Methanobrevibacter* sp. RUG833	2.5	73	16	1743	187745	189487	9.17	Methanogenic	Scotland	OMSG01000001.1	6	-
			15	6999	40007	47005	2.14			OMSG01000002.1	12	-
*Methanosarcina mazei* 1.H.A.2.3	4	68	12	2884	647	3530	4.16	Methanogenic	USA	JJQM01000083.1	-	-
*Methanosarcina mazei* 1.H.A.2.6	4	69	12	2759	531	3289	4.34	Methanogenic	USA	JJQN01000165.1	-	-

#: pseudogenes/introns, number of these elements in the tRNA gene clusters.

*tRNA gene clusters in archaea mobilome* - Since many genes surrounding the tRNA gene clusters are related to lateral gene transfer and the GC content of some tRNA gene cluster regions contrast with the genomic GC content [Supplementary data (Table V)], we surveyed the contigs in order to find sequences related to mobile genetic elements. Among the methanogens, a quite discrepancy intra and inter tRNA gene cluster region GC content could be observed, but none evidence of mobile genetic elements presence was obtained.

By the other hand, in the *Haloarcula californiae* ATCC 33799 strain there is a ~51 kb length contig (access number AOLS01000072.1) harboring the tRNA gene cluster and protein-coding genes with amino acid sequence similarity to Relaxase (identity 94% and coverage 100%) and conjugative TraD proteins (identity 95% and coverage 100%) present in the pNYT1 plasmid from *Haloarcula taiwanensis* strain Taiwanensis. In addition, it also presents a type II toxin-antitoxin system from hicA/B family. In *Halorubrum sp.* BV1, a contig with ~188 kb (access number JQKV01000005.1) also presented similarity with sequences from many archaea plasmids, showing coverage of 30% and identity of 96% with *Halorubrum trapanicum* plasmid pCBA1232-02. Besides that, the *in-silico* analysis determined the circular topology to this contig, characterising it as a replicon. Considering all these evidences, it seems that some archaea plasmids can be carrying tRNA gene clusters. Interestingly, some contigs harboring tRNA gene clusters also presented features common to archaea plasmids that could represent an ancestral presence of a plasmid.

Besides the plasmids, we performed a screening for provirus. VirSorter analysis detected putative viral sequences encompassing the regions of the tRNA gene clusters in two genomes, *H. vallismortis* ATCC 29715 and *Halorientalis* sp. IM101 [Supplementary data (Table VI)].

## DISCUSSION

Shared traits between prokaryotes and eukaryotes are helpful in the understanding of the tree of life evolution. tRNA gene cluster, a particular feature in RNA gene organisation, has been demonstrated to be prevalent in bacteria and eukaryotes, but there is a gap concerning its occurrence in archaea. Despite the significant presence in the *Bacteria* and *Eukarya* domains, the role of the tRNA gene cluster is under debate. In *Bacteria*, it has been implicated with a faster cell growing and in the modulation of the tRNA transcription and translation process.^20^ In contrast, some studies have not observed any improvement in the fitness of the organism by tRNA gene clusters. In addition, some of the genes from these clusters would be inactivated.^21^ In eukaryotes, there is a negative correlation between clusters of tRNA genes and chromosomal stability, since they can act as barriers to DNA replication and the consequent formation of genomic fragile sites.^5^ Besides that, the tRNA-derived fragments (tsRNAs or tRFs), identified in the three domains of life,^22^ could be generated from the tRNA gene clusters, being another common trait between prokaryotes and eukaryotes. The presence of tRNA gene clusters in the three domains of life reveals a common type of tRNA gene organisation, and as the function of these tRNA genes seems not be canonical, these clusters may be presenting a different functionality.

In this study, based on analyses of complete and draft archaeal genomes we demonstrated the prevalence of tRNA gene clusters in archaea. Considering the four archaea supergroups (Asgard, DPANN, TACK, and Euryarchaeota),^23^ tRNA gene cluster occurs at least in two class (*Halobacteria* and *Methanobacteria*) from Euryarchaeota. In fact, there is a bias concerning the number of genomes and drafts from Euryarchaeota, and therefore it is not possible to assume that this trait is exclusive of this supergroup.

Similarly to what was found in bacteria,^6^ ^,^ ^7^ archaea tRNA gene clusters seem to favor tRNA isotype redundancy, just increasing the number of tRNA gene copies. However, there was a slight increase in the diversity of isoacceptors for few tRNA species and genomes. For most genomes, the tRNA isoacceptors identified in the clusters were redundant. Since the tRNA isoacceptor abundance determine the codon translation efficiency,^24^ this result suggests that there is a selective pressure against the diversification of the isoacceptors encoded in tRNA gene clusters, favoring the translation efficiency of certain codons in the archaea. Indeed, it was observed in *Halobacteria* class a biased codon usage which was related to translational speed.^2^ Another common trait between archaea and bacteria is the presence of protein-coding genes within some tRNA gene clusters. In archaea, most of tRNA gene clusters harbor less than 20 tRNA genes, while in bacteria there is a considerable number of tRNA gene clusters with more than 20 tRNA genes. Besides tRNA genes, there are also tRNA pseudogenes in the tRNA gene clusters from prokaryotes and eukaryotes,^5^ ^,^ ^21^ suggesting a compromised canonical function, or may be related to other functions.

Concerning the origin of the tRNA gene clusters, in bacteria, it was assumed that they originated from *Firmicutes* phylum through a horizontal gene transfer event.^6^ Indeed, there is evidence showing the association of the tRNA gene clusters with mobile genetic elements. In several bacteria species there are plasmids carrying tRNA gene clusters,^6^ ^,^ ^7^ and in archaea, we have evidence that some contigs harboring tRNA gene clusters are associated with homologous sequences of archaea plasmids (this work). Interestingly, one of these contigs presented a relaxase gene, which is rare trait in archaeal plasmids,^25^ but a common trait of conjugative bacterial plasmids. In fact, the BLAST analyses of the nucleotide/protein sequence of this relaxase revealed high similarity with archaeal relaxase, but not with bacterial relaxase. Moreover, tRNA gene clusters are abundant in mycobacteriophages,^7^ ^,^ ^8^ ^,^ ^26^ also occurring in some bacteriophages.^27^ So far, the tRNA gene cluster had been only identified in one haloarchaeal virus genome, being composed of 36 tRNA genes.^28^ Here, using VirSorter, it was raised an evidence of two archaea genomes presenting tRNA gene clusters in regions predicted as provirus sequences. This result suggests that, in archaea, as in bacteria, viruses play a role in the transfer of the tRNA gene clusters.

While most of the tRNA gene clusters were composed of archaeal-type tRNAs, those from *M. mazei* genomes were bacterial-type and presented associated genes with high similarity to bacteria and bacteriophages. One of these genes encodes for chloramphenicol O-acetyltransferase, which has already been reported in other methanogenic archaea species.^29^ These results would suggest an inter-domain horizontal gene transfer, however, we could not discard the possibility of contamination, since the contigs that harbor these tRNA gene clusters are small (~7/8 kb) and contain only bacterial sequences, preventing the discrimination of the insertion sites in the archaea genome. Although we could not state the occurrence of this inter-domain transfer, there have already been reports of horizontal gene transfer between *M. mazei* species and bacteria.^30^ ^,^ ^31^

Altogether, it is clear that the tRNA gene cluster is a common feature in the three domains of life, which has independently evolved in each domain. In addition, it appears to be associated with mobile elements in bacteria and archaea.
