# Supplementary material for: Exploring tRNA gene cluster in archaea
Source: Mem Inst Oswaldo Cruz. 2019 Jan 7;114:e180348. doi: 10.1590/0074-02760180348 (PMC6333295; doi:10.1590/0074-02760180348)
Supplement: Supplementary file 1 [file 1678-8060-mioc-114-e180348-s.pdf]

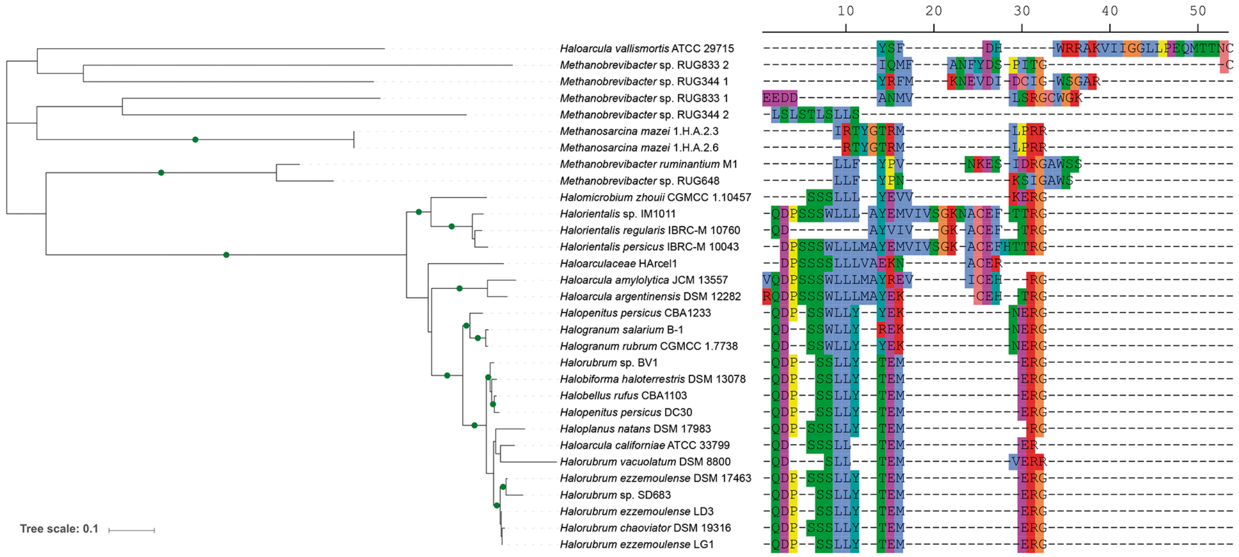

Fig. 1: maximum likelihood tree based on concatenated tRNA gene nucleotide sequences from tRNA gene clusters. In the right side, the tRNA isotype organisation (using the single letter amino acid code) is related with each tree branch. The gaps (- symbol) may not represent the actual distance between two adjacent tRNA genes, but the distance from the reference array. Each array group is depicted by the branch color. The green circles in the branches indicate bootstrap values ≥ 70.

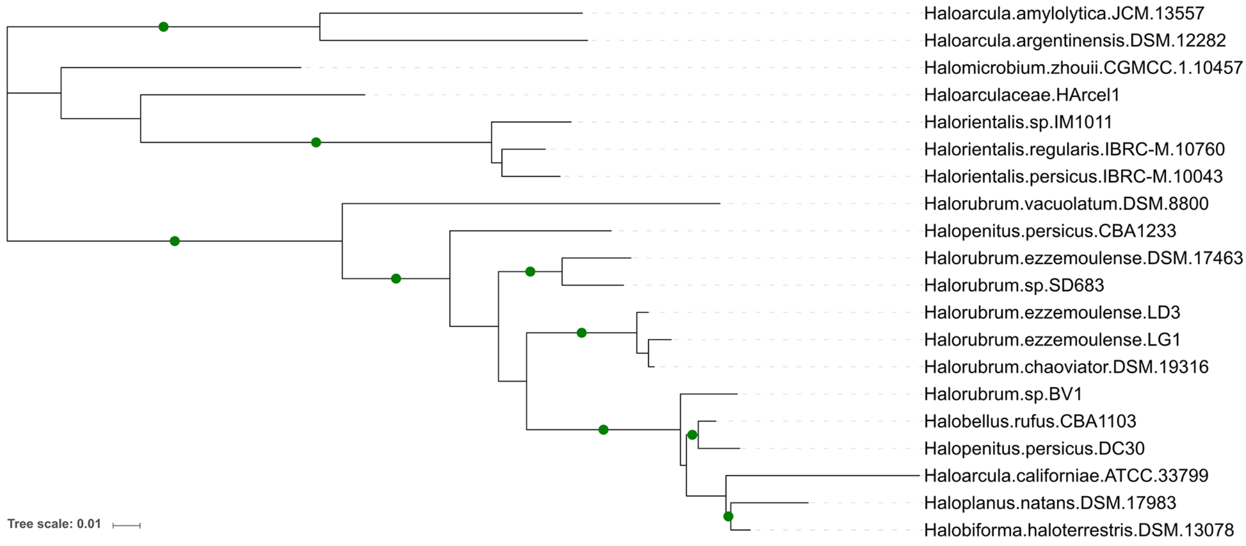

Fig. 2: maximum likelihood tree based on TROVE domain-containing protein sequences (511 aa) identified in the tRNA gene clusters. The green circles in the branches indicate bootstrap values ≥ 70.

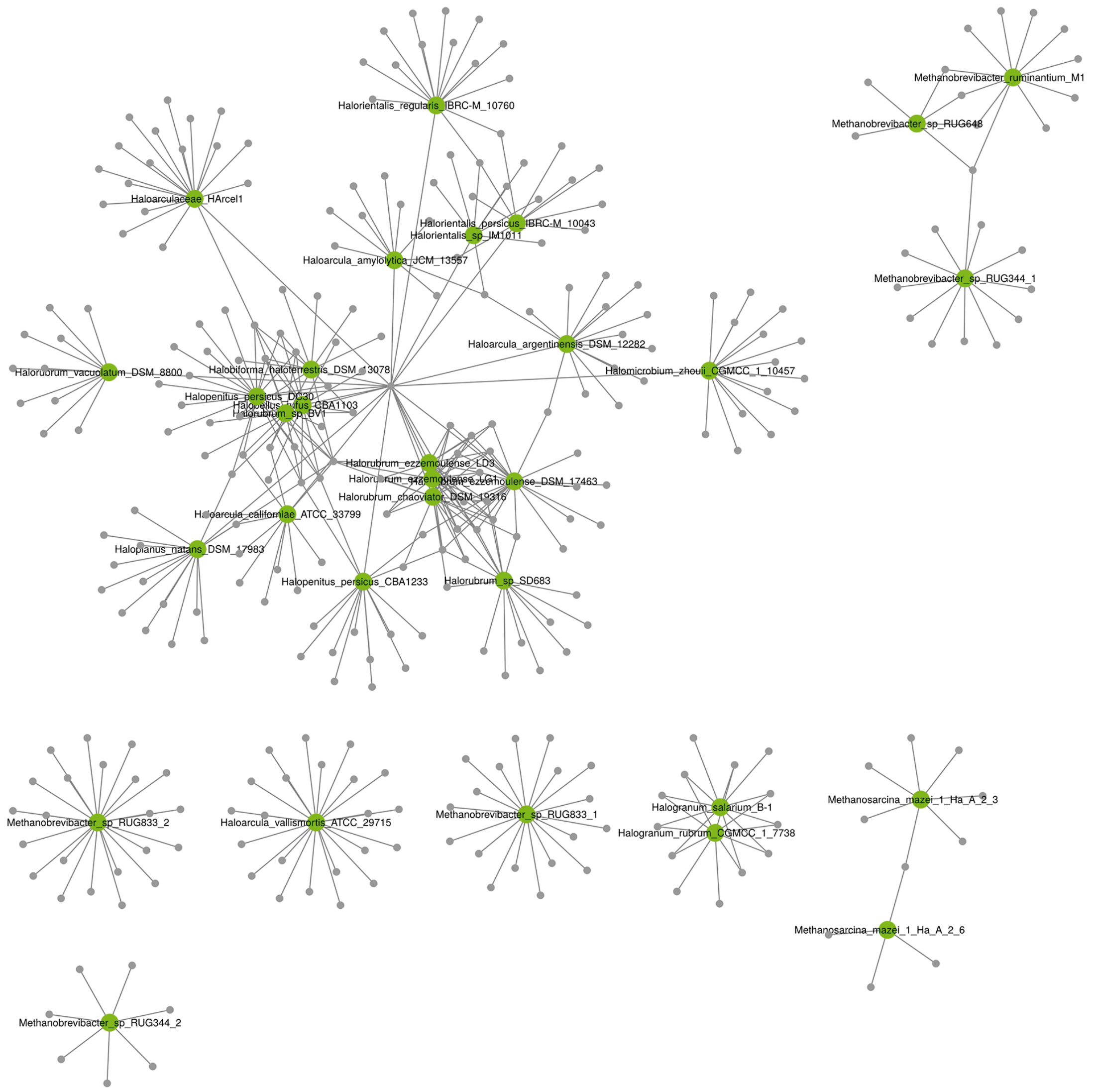

Fig. 3: the bipartite network of gene content associated to the tRNA gene clusters. Green and grey circles represent the tRNA gene clusters from the archaeal genomes and the genes associated to the tRNA gene clusters, respectively. Edges connect the tRNA gene clusters with the genes that it contains.

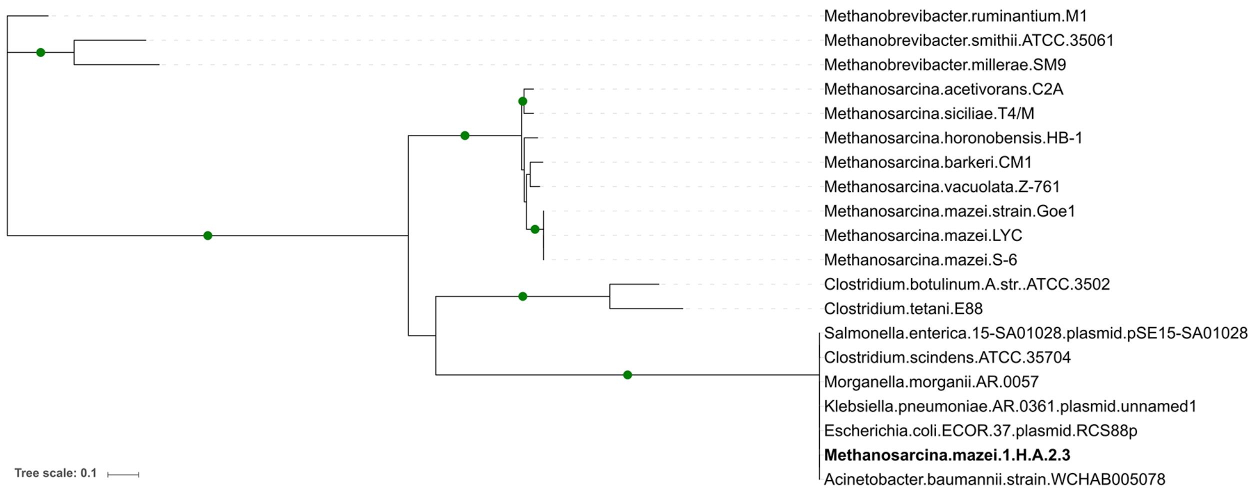

Fig. 4: maximum likelihood tree based on the chloramphenicol O-acetyltransferase gene sequences (665 bp). The green circles in the branches indicate bootstrap values ≥ 70.

TABLE I

tRNA gene copy number from genomes and tRNA gene clusters

	1		2		3		4		5		6		7		8		9		10		11		12		13		14		15	
	#G	#C	#G	#C	#G	#C	#G	#C	#G	#C	#G	#C	#G	#C	#G	#C	#G	#C	#G	#C	#G	#C	#G	#C	#G	#C	#G	#C	#G	#C
Ala	4	1	4	1	4	1	4	1	2	1	5	2	6	2	3	0	2	0	3	0	4	0	3	0	4	0	2	0	4	0
Gly	3	1	4	1	5	1	5	2	2	1	5	2	5	2	3	0	4	1	5	1	5	1	4	1	4	1	4	1	4	1
Pro	2	1	4	1	4	1	4	1	2	1	4	1	4	1	3	0	4	1	3	0	3	0	3	0	4	1	4	1	4	1
Thr	2	0	3	0	4	1	5	2	2	0	5	2	6	2	4	1	4	1	3	0	3	0	3	0	3	0	4	1	5	1
Val	4	1	4	2	3	0	5	1	1	0	5	2	5	2	3	0	3	0	4	0	4	0	5	2	3	0	3	0	3	0
Ser	6	3	7	3	7	3	5	1	4	2	8	4	8	4	6	3	6	2	7	2	7	2	7	3	6	2	6	2	7	3
Arg	5	1	7	2	6	2	7	2	3	0	6	1	6	1	6	1	5	1	7	1	7	2	5	1	7	1	6	1	6	1
Leu	6	2	8	3	8	3	7	2	5	2	8	3	8	3	7	2	8	2	7	2	7	2	8	3	7	2	7	2	8	2
Phe	2	1	1	0	1	0	2	1	2	1	2	1	2	1	1	0	1	0	1	0	1	0	1	0	1	0	1	0	1	0
Asn	2	1	1	0	2	0	2	1	2	1	2	1	1	0	1	0	1	0	3	1	3	1	1	0	2	1	1	0	1	0
Lys	3	1	2	0	3	1	3	1	2	1	4	1	3	1	2	0	2	0	3	1	3	1	3	1	3	1	2	0	2	0
Asp	2	1	3	1	3	1	1	1	1	0	2	1	2	1	3	1	3	1	4	1	4	1	1	0	3	1	3	1	2	1
Glu	2	1	4	2	4	2	3	1	1	0	4	2	4	2	4	2	4	2	4	2	4	2	4	2	4	2	5	2	4	1
His	1	0	2	1	2	1	2	1	1	0	1	0	2	1	1	0	1	0	1	0	1	0	1	0	1	0	1	0	1	0
Gln	2	0	3	1	3	1	3	1	1	0	3	1	2	0	3	1	3	1	3	1	3	1	2	0	3	1	3	1	3	1
Ile	2	1	2	1	1	0	3	2	2	1	2	1	2	1	1	0	1	0	1	0	1	0	1	0	1	0	1	0	1	0
Met	2	0	3	1	3	1	3	1	2	0	3	1	5	2	3	1	3	1	3	0	3	0	2	0	2	0	3	1	5	1
Tyr	2	1	2	1	2	1	2	1	2	1	2	1	2	1	1	0	2	1	3	1	3	1	2	1	2	1	2	1	2	1
Cys	1	0	2	1	2	1	2	1	1	0	2	1	2	1	1	0	1	0	1	0	1	0	1	0	1	0	1	0	1	0
Trp	2	1	2	1	2	1	2	1	2	1	3	1	2	1	1	0	1	0	2	1	2	1	1	0	2	1	1	0	1	0

	16		17		18		19		20		21		22		23		24		25		26		27		28			29		
	#G	#C	#G	#C	#G	#C	#G	#C	#G	#C	#G	#C	#G	#C	#G	#C	#G	#C	#G	#C	#G	#C	#G	#C	#G	#C1	#C2	#G	#C1	#C2
Ala	5	2	3	0	2	0	2	0	3	0	4	0	2	0	3	0	2	0	3	0	5	2	3	0	2	1	0	3	1	1
Gly	6	2	5	1	5	1	5	1	5	1	5	1	5	1	4	0	6	1	6	1	3	0	4	1	4	2	0	3	2	1
Pro	3	0	4	1	4	1	4	1	4	1	4	1	4	1	3	0	4	1	4	1	4	1	4	1	1	0	0	3	0	1
Thr	4	1	4	1	4	1	4	1	5	1	4	1	4	1	4	1	5	2	5	2	3	0	3	1	4	0	1	4	0	1
Val	4	2	3	0	3	0	3	0	3	0	3	0	3	0	4	1	4	0	4	0	4	1	3	0	3	1	0	3	1	0
Ser	4	0	7	3	7	3	6	2	7	2	6	2	6	2	5	1	4	0	4	0	7	3	6	2	7	1	4	5	1	1
Arg	6	1	6	1	6	1	6	1	7	1	6	1	6	1	6	1	9	4	9	4	6	1	5	1	6	2	0	6	1	0
Leu	5	0	7	2	7	2	7	2	10	2	7	2	8	2	8	2	7	1	7	1	8	3	7	2	9	0	5	7	1	0
Phe	2	1	1	0	1	0	1	0	3	0	1	0	1	0	1	0	2	0	2	0	1	0	2	0	2	1	0	3	0	2
Asn	1	0	1	0	1	0	1	0	2	0	1	0	1	0	1	0	2	0	2	0	2	1	0	0	2	1	0	4	1	1
Lys	4	1	2	0	2	0	2	0	2	0	2	0	2	0	2	0	2	0	2	0	3	1	2	0	2	1	0	3	1	0
Asp	2	1	3	1	2	1	3	1	2	1	3	1	3	1	3	1	1	0	1	0	3	1	3	1	3	2	0	4	2	1
Glu	3	1	4	2	4	2	4	2	4	2	4	2	4	2	4	2	3	0	3	0	4	2	6	2	2	1	0	3	2	0
His	1	0	1	0	1	0	1	0	1	0	1	0	1	0	1	0	1	0	1	0	1	0	1	0	1	0	0	1	0	0
Gln	3	1	3	1	3	1	2	1	3	1	3	1	3	1	3	1	2	0	2	0	2	0	3	1	2	0	0	3	0	1
Ile	2	1	1	0	1	0	1	0	1	0	1	0	1	0	1	0	2	1	2	0	1	0	1	0	2	1	0	5	0	2
Met	3	0	3	1	3	1	3	1	3	1	3	1	3	1	3	1	4	1	4	1	2	0	2	1	4	1	0	4	1	1
Tyr	2	1	2	1	2	1	2	1	2	1	2	1	2	1	1	0	2	1	2	1	1	0	3	1	2	1	0	3	0	1
Cys	2	1	1	0	1	0	1	0	2	0	1	0	1	0	1	0	3	0	3	0	2	1	1	0	2	1	0	3	1	1
Trp	1	0	1	0	1	0	1	0	1	0	1	0	1	0	1	0	1	0	1	0	1	0	1	0	2	1	0	1	1	0

#G: number of genes coded by the whole genome; #C, number of genes coded by the tRNA gene cluster. The numbers in the red block represent the amino acids only coded by the tRNA gene cluster. The upper numbers represent the genomes: 1, *Methanobrevibacter ruminantium* M1; 2, *Haloarcula amylolytica* JCM 13557; 3, *Haloarcula argentinensis* DSM 12282; 4, *Haloarcula vallismortis* ATCC 29715; 5, *Methanobrevibacter* sp. RUG648; 6, Halorientalis sp. IM1011; 7, *Halorientalis persicus* IBRC-M 10043; 8, *Haloarcula californiae* ATCC33799; 9, *Halobiforma haloterrestris* DSM13078; 10, *Halogranum rubrum* CGMCC1 7738; 11, *Halogranum salarium* B1; 12, *Halomicrobium zhouii* CGMCC1 10457; 13, *Halopenitus persicus* CBA1233; 14, *Halopenitus persicus* DC30; 15, *Haloplanus natans* DSM17983; 16, *Halorientalis regularis* IBRCM10760; 17, *Halorubrum chaoviator* DSM19316; 18, *Halorubrum ezzemoulense* DSM17463; 19, *Halorubrum ezzemoulense* LD3; 20, *Halorubrum ezzemoulense* LG1; 21, *Halorubrum* sp. BV1; 22, *Halorubrum* sp. SD683; 23, *Halorubrum vacuolatum* DSM8800; 24, *Methanosarcina mazei* 1.H.A.2.3; 25, *Methanosarcina mazei* 1.H.A.2.6; 26, *Haloarculaceae archaeon* HArcell1; 27, *Halobellus rufus* CBA1103; 28, *Methanobrevibacter* sp. RUG344; 29, *Methanobrevibacter* sp. RUG833.

TABLE II

Different isoacceptors provided by the clusters

Genomes	anticodon	#
Haloarcula amylolytica JCM 13557	cac	2
Haloarcula argentinensis DSM 12282	tcg	2
Haloarcula californiae ATCC 33799	tga	1
Haloarcula vallismortis ATCC 29715	gtc	1
Halobellus rufus CBA1103	tgt	1
Methanobrevibacter sp. RUG344	aag	1
	tgc	1
	tga	2
	caa	1
Methanobrevibacter sp. RUG648	tgc	1
	tga	1
	caa	1
Methanobrevibacter sp. RUG833	acc	1
	acg	1
	aaa	1
	ctt	1
	tga	1
	tgc	2
	tcc	2
	cca	1

TABLE III

Genomic context of the tRNA gene clusters

Genome	Putative protein
Methanobrevibacter ruminantium M1	Acyltransferase family protein
	phage holin family protein
	hypothetical protein
	TatD family deoxyribonuclease
	tRNA threonylcarbamoyladenosine dehydratase
	pyridoxamine 5’-phosphate oxidase
	adenylosuccinate lyase
	CPBP family intramembrane metalloprotease
	CPBP family intramembrane metalloprotease
	transcriptional regulator
	hypothetical protein
	hypothetical protein
	hypothetical protein
	hypothetical protein
	hypothetical protein
	CPBP family intramembrane metalloprotease
Halorubrum vacuolatum DSM 8800	ATP-binding protein
	hypothetical protein
	SWIM zinc finger domain-containing protein
	hypothetical protein
	hypothetical protein
	hypothetical protein
	transcriptional regulator TrmB
	growth inhibitor
	MarR family transcriptional regulator
	pirin family protein
	hypothetical protein
	TROVE domain-containing protein

Genome	Putative protein
	hypothetical protein
	hypothetical protein
	hypothetical protein
	ATP-binding protein
	hypothetical protein
	NurA domain-containing protein
	SMC family ATPase
Halorubrum sp. SD683	hypothetical protein
	hypothetical protein
	hypothetical protein
	VanZ family protein
	hypothetical protein
	nucleotide sugar dehydrogenase
	UTP--glucose-1-phosphate uridylyltransferase
	hypothetical protein
	hypothetical protein
	transposase, IS605 OrfB family
	TROVE domain-containing protein
	hypothetical protein
	hypothetical protein
	nucleotidyltransferase domain-containing protein
	AbrB/MazE/SpoVT family DNA-binding domain
	type II toxin-antitoxin system VapC family toxin
	hypothetical protein
	nucleotidyltransferase
	hypothetical protein
	hypothetical protein
	hypothetical protein
	PIN domain-containing protein
	AbrB/MazE/SpoVT family DNA-binding domain
	hypothetical protein
	hypothetical protein
	plasmid replication protein RepH
Halorubrum sp. BV1	TATA-box-binding protein
	hypothetical protein
	hypothetical protein
	hypothetical protein
	hypothetical protein
	transcriptional regulator
	hypothetical protein
	hypothetical protein
	hypothetical protein
	hypothetical protein
	TROVE domain-containing protein
	hypothetical protein
	Lrp/AsnC family transcriptional regulator
	hypothetical protein
	Orc1-type DNA replication protein
Halorubrum ezzemoulense LG1	VanZ family protein
	hypothetical protein
	nucleotide sugar dehydrogenase
	hypothetical protein
	transcriptional regulator
	UTP--glucose-1-phosphate uridylyltransferase
	hypothetical protein
	hypothetical protein

Genome	Putative protein
	UTP--glucose-1-phosphate uridylyltransferase
	hypothetical protein
	hypothetical protein
	hypothetical protein
	hypothetical protein
	transposase, IS605 OrfB family
	hypothetical protein
	TROVE domain-containing protein
	hypothetical protein
	hypothetical protein
	hypothetical protein
	hypothetical protein
	hypothetical protein
Halorubrum ezzemoulense LD3	VanZ family protein
	hypothetical protein
	nucleotide sugar dehydrogenase
	hypothetical protein
	transcriptional regulator
	UTP--glucose-1-phosphate uridylyltransferase
	hypothetical protein
	hypothetical protein
	UTP--glucose-1-phosphate uridylyltransferase
	hypothetical protein
	hypothetical protein
	hypothetical protein
	hypothetical protein
	transposase, IS605 OrfB family
	hypothetical protein
	TROVE domain-containing protein
	hypothetical protein
	hypothetical protein
	hypothetical protein
	hypothetical protein
Halorubrum ezzemoulense DSM 17463	VanZ family protein
	hypothetical protein
	nucleotide sugar dehydrogenase
	hypothetical protein
	transcriptional regulator
	UTP--glucose-1-phosphate uridylyltransferase
	hypothetical protein
	hypothetical protein
	UTP--glucose-1-phosphate uridylyltransferase
	hypothetical protein
	hypothetical protein
	hypothetical protein
	hypothetical protein
	transposase, IS605 OrfB family
	hypothetical protein
	TROVE domain-containing protein
	hypothetical protein
	hypothetical protein
	hypothetical protein
	hypothetical protein
	DNA polymerase subunit beta
	IS5/IS1182 family transposase
Halorubrum chaoviator DSM 19316	hypothetical protein
	VanZ family protein

Genome	Putative protein
	hypothetical protein
	nucleotide sugar dehydrogenase
	hypothetical protein
	hypothetical protein
	UTP--glucose-1-phosphate uridylyltransferase
	hypothetical protein
	hypothetical protein
	hypothetical protein
	hypothetical protein
	transposase, IS605 OrfB family
	TROVE domain-containing protein
	hypothetical protein
	hypothetical protein
	hypothetical protein
	hypothetical protein
	hypothetical protein
	transposase, IS605 OrfB family
	DNA polymerase beta
	hypothetical protein
	transposase, IS605 OrfB family protein
	hypothetical protein
	PadR family transcriptional regulator
	nucleotidyltransferase domain protein
	hypothetical protein
	hypothetical protein
Halorientalis sp. IM1011	hypothetical protein
	chromosome condensation protein CrcB
	fluoride efflux transporter CrcB
	AmiS/UreI transporter
	Chaperonin GroEL (HSP60 family)
	acetamidase/formamidase family protein
	hypothetical protein
	carboxymuconolactone decarboxylase family protein
	AMP-dependent synthetase
	hypothetical protein
	hypothetical protein
	TROVE domain-containing protein
	ATP-binding protein
	CopG family transcriptional regulator
	transcriptional regulator
	transcriptional regulator
	hypothetical protein
	ArsR family transcriptional regulator
	hypothetical protein
Halorientalis regularis IBRC-M 10760	AMP-dependent synthetase
	bleomycin resistance protein
	bacterio-opsin activator
	hypothetical protein
	acetone carboxylase subunit alpha
	CBS domain-containing protein
	hypothetical protein
	hypothetical protein
	HNH endonuclease
	DNA-binding protein
	hypothetical protein
	hypothetical protein
	TROVE domain-containing protein

Genome	Putative protein
	IS200/IS605 family transposase
	hypothetical protein
	hypothetical protein
	transcriptional regulator
	transcriptional regulator
	integrase
Halorientalis persicus IBRC-M 10043	hypothetical protein
	HEAT repeat-containing protein
	VWA domain-containing protein
	hypothetical protein
	ATP-binding protein
	HSP20 family protein
	type II toxin-antitoxin system VapC family toxin
	hypothetical protein
	hypothetical protein
	TROVE domain-containing protein
	hypothetical protein
	hypothetical protein
	hypothetical protein
	5’/3’-nucleotidase SurE
	hypothetical protein
	ORC complex protein Cdc6/Orc1
Haloplanus natans DSM 17983	hypothetical protein
	hypothetical protein
	hypothetical protein
	hypothetical protein
	transposase
	hypothetical protein
	hypothetical protein
	hypothetical protein
	PadR family transcriptional regulator
	IS1341-type transposase
	transcriptional regulator
	HNH endonuclease
	transposase
	TROVE domain-containing protein
	hypothetical protein
	SWIM zinc finger domain-containing protein
	type II toxin-antitoxin system death-on-curing family
	hypothetical protein
	hypothetical protein
	MarR family transcriptional regulator
	Mrr restriction system protein
	IS200/IS605 family transposase
	transposase
	Orc1-type DNA replication protein
Halopenitus persicus CBA 1233	hypothetical protein
	hypothetical protein
	type IV secretory system conjugative DNA transfer family protein
	hypothetical protein
	hypothetical protein
	hypothetical protein
	hypothetical protein
	hypothetical protein
	TROVE domain-containing protein
	hypothetical protein
	hypothetical protein

Genome	Putative protein
	hypothetical protein
	transcription initiation factor IIB family protein
	hypothetical protein
	hypothetical protein
	ArsR family transcriptional regulator
	hypothetical protein
	metallophosphatase family protein
	resolvase
	hypothetical protein
	hypothetical protein
Halopenitus persicus DC30	hypothetical protein
	DoxX family protein
	Na+-dependent transporter
	Zn-dependent oxidoreductase
	transcriptional regulator
	DoxX family protein
	hypothetical protein
	hypothetical protein
	transcriptional regulator
	ArsR family transcriptional regulator
	hypothetical protein
	PadR family transcriptional regulator
	transcriptional regulator
	hypothetical protein
	hypothetical protein
	hypothetical protein
	hypothetical protein
	TROVE domain-containing protein
	hypothetical protein
	Lrp/AsnC family transcriptional regulator
	hypothetical protein
	Orc1-type DNA replication protein
	hypothetical protein
	hypothetical protein
	restriction endonuclease
	hypothetical protein
	AI-2E family transporter
	hypothetical protein
	AI-2E family transporter
	hypothetical protein
	hypothetical protein
	hypothetical protein
	hypothetical protein
	hypothetical protein
Halomicrobium zhouii CGMCC1 10457	hypothetical protein
	hypothetical protein
	SWIM zinc finger family protein
	MFS transporter
	hypothetical protein
	hypothetical protein
	hypothetical protein
	hypothetical protein
	bacterio-opsin activator
	hypothetical protein
	hypothetical protein
	hypothetical protein
	sensor histidine kinase

Genome	Putative protein
	PIN domain protein
	PIN domain-containing protein
	hypothetical protein
	IS630 family transposase
	TROVE domain-containing protein
	hypothetical protein
	hypothetical protein
	hypothetical protein
	hypothetical protein
	hypothetical protein
	hypothetical protein
	hypothetical protein
	hypothetical protein
	hypothetical protein
	hypothetical protein
Halogranum salarium B1	hypothetical protein
	aminoglycoside phosphotransferase family protein
	Bacterio-opsin activator HTH domain protein
	cytochrome P450
	transcriptional regulator, TrmB
	hypothetical protein
	hypothetical protein
	hypothetical protein
	ArsR family transcriptional regulator
	hypothetical protein
	hypothetical protein
	hypothetical protein
	hypothetical protein
	conjugal transfer protein
	hypothetical protein
	transfer complex protein
Halogranum rubrum CGMCC1 7738	DNA-binding transcriptional regulator
	homoserine O-acetyltransferase
	FAD-binding protein
	hypothetical protein
	hypothetical protein
	conjugal transfer protein
	hypothetical protein
	hypothetical protein
	hypothetical protein
	hypothetical protein
	hypothetical protein
	ArsR family transcriptional regulator
	hypothetical protein
	hypothetical protein
	hypothetical protein
	SWIM zinc finger domain-containing protein
	hypothetical protein
	hypothetical protein
	zinc ribbon domain-containing protein
	hypothetical protein
	nucleotidyltransferase domain-containing protein
	MBL fold hydrolase
	hypothetical protein
Halobiforma haloterrestris DSM 13078	hypothetical protein
	hypothetical protein

Genome	Putative protein
	hypothetical protein
	hypothetical protein
	DNA polymerase I
	Orc1-type DNA replication protein
	hypothetical protein
	Lrp/AsnC family transcriptional regulator
	hypothetical protein
	TROVE domain-containing protein
	transposase, IS605 OrfB family
	hypothetical protein
	hypothetical protein
	ArsR family transcriptional regulator
	transposase
	transcriptional regulator
	hypothetical protein
	MarR family transcriptional regulator
	hypothetical protein
	hypothetical protein
	hypothetical protein
	hypothetical protein
Haloarcula vallismortis ATCC 29715	hypothetical protein
	hypothetical protein
	DNA cytosine methyltransferase
	DNA (cytosine-5-)-methyltransferase
	hypothetical protein
	base excision DNA repair protein
	hypothetical protein
	MarR family transcriptional regulator
	hypothetical protein
	hypothetical protein
	hypothetical protein
	hypothetical protein
	hypothetical protein
	hypothetical protein
	transposase, IS605 OrfB family protein
	hypothetical protein
	hypothetical protein
	hypothetical protein
	hypothetical protein
	hypothetical protein
	hypothetical protein
	hypothetical protein
	hypothetical protein
	hypothetical protein
	hypothetical protein
	hypothetical protein
	hypothetical protein
	ArsR family transcriptional regulator
	hypothetical protein
	hypothetical protein
	hypothetical protein
	hypothetical protein
	hypothetical protein
	ATP-binding protein
	hypothetical protein
Haloarcula californiae ATCC 33799	hypothetical protein
	hypothetical protein

Genome	Putative protein
	relaxase/mobilization nuclease domain-containing protein
	conjugative transfer protein TraD
	hypothetical protein
	hypothetical protein
	hypothetical protein
	hypothetical protein
	hypothetical protein
	TROVE domain-containing protein
	hypothetical protein
	hypothetical protein
	HTH domain-containing protein
	hypothetical protein
	hypothetical protein
	hypothetical protein
	sugar-specific transcriptional regulator TrmB
	hypothetical protein
	hypothetical protein
	hypothetical protein
	hypothetical protein
Haloarcula argentinensis DSM 12282	Asp-tRNA(Asn)/Glu-tRNA(Gln) amidotransferase
	Asp-tRNA(Asn)/Glu-tRNA(Gln) amidotransferase
	protease HtpX
	hypothetical protein
	glycosyltransferase family 4 protein
	MFS transporter
	sugar transferase
	ribonuclease P
	hypothetical protein
	mechanosensitive ion channel family protein
	ADP-ribosyl-[dinitrogen reductase] hydrolase
	hypothetical protein
	TROVE domain-containing protein
	hypothetical protein
	hypothetical protein
	hypothetical protein
	hypothetical protein
	AMP-dependent synthetase
	class I SAM-dependent methyltransferase
	fumarylacetoacetate hydrolase
	IclR family transcriptional regulator
	hypothetical protein
	hypothetical protein
	cytochrome b/b6 domain-containing protein
	hypothetical protein
	4Fe-4S dicluster domain-containing protein
Haloarcula amylolytica JCM 13557	Type I phosphodiesterase / nucleotide pyrophosphatase
	IS5/IS1182 family transposase
	IS4/IS5 family transposase
	transposase (ISH3)
	hypothetical protein
	VWA domain-containing protein
	ATP-binding protein
	hypothetical protein
	hypothetical protein
	Sigma-70, region 4
	TROVE domain-containing protein
	hypothetical protein
	hypothetical protein

Genome	Putative protein
	MBL fold metallo-hydrolase
	NADH-quinone oxidoreductase subunit N
	NADH-quinone oxidoreductase subunit M
	NADH-quinone oxidoreductase subunit L
	NADH-quinone oxidoreductase
	proton-conducting membrane transporter
	NADH dehydrogenase subunit J
	NADH dehydrogenase
	NADH dehydrogenase subunit H
Methanosarcina mazei 1.A.H.2.3	hypothetical protein
	integrase
	chloramphenicol O-acetyltransferase
	tetracycline resistance protein
	lysozyme
	MFS transporter
	type VI secretion protein
Methanosarcina mazei 1.A.H.2.6	chloramphenicol acetyltransferase
	integrase
	hypothetical protein
	lac operon repressor
Haloarculaceae Harcel1	DNA polymerase I
	hypothetical protein
	RNA polymerase subunit sigma-70
	PadR family transcriptional regulator
	hypothetical protein
	TROVE domain-containing protein
	hypothetical protein
	hypothetical protein
	hypothetical protein
	hypothetical protein
	hypothetical
	type II toxin-antitoxin system VapC family toxin
	hypothetical protein
	orc1/cdc6 family replication initiation protein
	orc1/cdc6 family replication initiation protein
	MarR family transcriptional regulator
Halobellus rufus CBA1103	cell division control protein Cdc6
	HNH endonuclease
	hypothetical protein
	MarR family transcriptional regulator
	hypothetical protein
	TROVE domain-containing protein
	hypothetical protein
	hypothetical protein
	uncharacterized protein NP_6024A
	hypothetical protein
	transcriptional regulator
	PadR family transcriptional regulator
	hypothetical protein
	hypothetical protein
Methanobrevibacter sp. RUG833#1	hypothetical protein
	hypothetical protein
	ATPase
	hypothetical protein
	hypothetical protein
	hypothetical protein
	hypothetical protein

Genome	Putative protein
	hypothetical protein
	hypothetical protein
	hypothetical protein
	hypothetical protein
	hypothetical protein
	hypothetical protein
	hypothetical protein
	hypothetical protein
	hypothetical protein
	hypothetical protein
	hypothetical protein
Methanobrevibacter sp. RUG833#2	hypothetical protein
	hypothetical protein
	hypothetical protein
	hypothetical protein
	hypothetical protein
	hypothetical protein
	hypothetical protein
	hypothetical protein
	Uncharacterised protein
	Uncharacterised protein
	hypothetical protein
	hypothetical protein
	NUDIX hydrolase
	hypothetical protein
	uncharacterized protein DUF2829
	hypothetical protein
	hypothetical protein
	hypothetical protein
	hypothetical protein
	class I glutamine amidotransferase-like protein
	putative COOH-NH2 ligase
	glucosamine-fructose-6-phosphate aminotransferase
Methanobrevibacter sp. RUG344#1	hypothetical protein
	hypothetical protein
	hypothetical protein
	TetR/AcrR family transcriptional regulator
	N-acetyltransferase
	flavodoxin family protein
	pyridoxamine 5’-phosphate oxidase family protein
	hypothetical protein
	MULTISPECIES: MFS transporter
	MULTISPECIES: MFS transporter
	MarR family transcriptional regulator
	VWA domain-containing protein
	MULTISPECIES: hypothetical protein
Methanobrevibacter sp. RUG344#2	ABC transporter
	ABC transporter ATP-binding protein
	ABC transporter permease
	cell division protein FtsX
	hypothetical protein
	MULTISPECIES: DUF4012 domain-containing protein
	DUF2124 domain-containing protein

The red labels indicate the putative proteins encoded within the tRNA gene clusters.

TABLE IV

Blast results for the genes associated with the tRNA gene clusters from *Methanosarcina mazei* strains

Hits	Cover	E-value	Ident
***Methanosarcina mazei* 1.H.A.2.3**			
MULTISPECIES: hypothetical protein [Bacteria][Archaea]	100,00%	1,00E-38	100,00%
hypothetical protein DU74_04035 [*Methanosarcina mazei*]	100,00%	1,00E-38	100,00%
hypothetical protein A0256_24455 [*Mucilaginibacter* sp. PAMC 26640]	46,00%	2,00E-12	100,00%
tetracycline resistance protein class C [*Salmonella enterica* subsp. enterica serovar Enteritidis]	37,00%	2,00E-07	100,00%
hypothetical protein T03_6065 [*Trichinella britovi*]	46,00%	6,00E-07	84,00%
phosphoribosylaminoimidazole carboxylase [*Brucella melitensis*]	33,00%	3,00E-05	100,00%
phosphoribosylaminoimidazole carboxylase [Streptomyces sp. Ru72]	33,00%	3,00E-05	100,00%
hypothetical protein [*Bacillus cereus*]	33,00%	8,00E-05	100,00%
ATP-grasp domain-containing protein [*Streptococcus equinus*]	33,00%	1,00E-04	100,00%
ATP-grasp domain-containing protein [*Prevotella nigrescens*]	33,00%	2,00E-04	100,00%
integrase [*Methanosarcina mazei*]	74,00%	8,00E-102	100,00%
integrase [*Escherichia coli*]	58,00%	4,00E-79	100,00%
phage integrase family protein [*Escherichia coli*]	58,00%	1,00E-78	100,00%
site-specific integrase [*Escherichia coli*]	58,00%	2,00E-78	100,00%
site-specific integrase [*Escherichia coli*]	58,00%	3,00E-78	100,00%
site-specific integrase [*Escherichia coli*]	58,00%	6,00E-78	99,00%
site-specific integrase [*Escherichia coli*]	58,00%	7,00E-78	99,00%
integrase [*Escherichia coli*]	58,00%	7,00E-78	100,00%
site-specific integrase [*Escherichia coli*]	58,00%	9,00E-78	99,00%
phage integrase family protein [*Escherichia coli* 2875000]	58,00%	9,00E-78	99,00%
chloramphenicol O-acetyltransferase [(Clostridium) scindens ATCC 35704]	100,00%	1,00E-166	100,00%
CmR [MISSA donor vector pL-ccdB]	100,00%	2,00E-166	100,00%
chloramphenicol acetyltransferase [*Escherichia coli*]	100,00%	3,00E-166	100,00%
chloramphenicol acetyltransferase [*Escherichia coli*]	100,00%	3,00E-166	100,00%
chloramphenicol acetyltransferase [*Escherichia coli* LAU-EC8]	100,00%	4,00E-166	100,00%
MULTISPECIES: type A-1 chloramphenicol O-acetyltransferase [Bacteria][Archaea]	100,00%	4,00E-166	100,00%
type A-1 chloramphenicol O-acetyltransferase [*Photobacterium damselae*]	100,00%	5,00E-166	99,00%
chloramphenicol O-acetyltransferase, partial [*Blautia obeum* ATCC 29174]	100,00%	1,00E-165	99,00%
type A-1 chloramphenicol O-acetyltransferase [*Escherichia coli*]	100,00%	1,00E-165	99,00%
chloramphenicol acetyltransferase [synthetic construct]	100,00%	1,00E-165	99,00%
tetracycline resistance protein [*Methanosarcina mazei*]	100,00%	8,00E-98	100,00%
tetracycline resistance protein [*Methanosarcina mazei*]	100,00%	5,00E-97	99,00%
MFS_1 [uncultured bacterium]	68,00%	2,00E-60	100,00%
tetracycline-efflux transporter [Shuttle vector pKV12]	76,00%	2,00E-60	92,00%
MFS_1 [uncultured bacterium]	68,00%	2,00E-60	100,00%
tetracycline resistance protein, class C (TetA(C)) [*Escherichia coli* R424]	68,00%	2,00E-60	100,00%
conserved hypothetical protein [*Brucella pinnipedialis* B2/94]	68,00%	2,00E-60	100,00%
MFS_1 [uncultured bacterium]	68,00%	2,00E-60	100,00%
MFS transporter [*Lamprocystis purpurea*]	68,00%	2,00E-60	100,00%
tetracycline resistance protein [*Escherichia coli* KD2]	68,00%	2,00E-60	100,00%
MULTISPECIES: lysozyme [Bacteria][Archaea]	100,00%	4,00E-110	100,00%
gene 3.5 [*Enterobacteria phage* T7]	100,00%	2,00E-109	99,00%
unnamed protein product [*Enterobacteria phage* T7]	100,00%	7,00E-109	99,00%
gene 3.5 [*Enterobacteria phage* T7]	100,00%	3,00E-108	99,00%
gp3.5 [*Enterobacteria phage* 13a]	100,00%	3,00E-107	97,00%
lysin, N-acetylmuramoyl-L-alanine amidase [*Escherichia phage* 64795_ec1]	100,00%	4,00E-107	97,00%
Chain A, The Structure Of Bacteriophage T7 Lysozyme, A Zinc Amidase And An Inhibitor Of T7 Rna Polymerase	96,00%	2,00E-105	100,00%
N-acetylmuramoyl-L-alanine amidase [*Enterobacteria phage* T3]	100,00%	5,00E-105	95,00%
lysin [*Escherichia phage* CICC 80001]	100,00%	7,00E-105	95,00%
endolysin [*Escherichia phage* HZ2R8]	100,00%	1,00E-104	95,00%
MFS transporter [*Methanosarcina mazei*]	100,00%	0.0	100,00%
MFS transporter [*Methanosarcina mazei*]	100,00%	0.0	99,00%
related to mfs-multidrug-resistance transporter [*Sporisorium reilianum* SRZ2]	100,00%	0.0	94,00%

tetracycline resistance protein [*Enterococcus faecalis* T1]	100,00%	0.0	95,00%
MULTISPECIES: tetracycline efflux MFS transporter Tet(C) [Bacteria]	100,00%	0.0	94,00%
Tet(A)/Tet(B)/Tet(C) family tetracycline efflux MFS transporter [*Escherichia coli*]	100,00%	0.0	94,00%
Tet(A)/Tet(B)/Tet(C) family tetracycline efflux MFS transporter [*Escherichia coli*]	100,00%	0.0	94,00%
Tetracycline efflux protein TetA [*Vibrio cholerae*]	100,00%	0.0	94,00%
tetracycline resistance protein, class C (TetA(C)) [*Escherichia coli* H299]	100,00%	0.0	94,00%
MFS transporter [*Lamprocystis purpurea*]	100,00%	0.0	94,00%
hypothetical protein CLOM621_09114 [*Clostridium* sp. M62/1]	100,00%	4,00E-22	100,00%
MULTISPECIES: type VI secretion protein [Bacteria][Archaea]	100,00%	5,00E-22	100,00%
type VI secretion protein [*Klebsiella pneumoniae*]	100,00%	5,00E-21	98,00%
hypothetical protein [*Escherichia coli*]	100,00%	1,00E-20	98,00%
hypothetical protein B7939_12685 [*Eggerthia catenaformis*]	73,00%	2,00E-12	94,00%
hypothetical protein LH128_23591 [*Sphingomonas* sp. LH128]	71,00%	2,00E-12	100,00%
conjugative transfer ATPase [*Pseudomonas* sp. WCHP16]	73,00%	5,00E-12	97,00%
conjugative transfer ATPase [*Acidovorax* sp. T1m]	73,00%	5,00E-12	97,00%
conjugative transfer ATPase [*Pseudomonas aeruginosa*]	73,00%	7,00E-12	97,00%
conjugative transfer ATPase [*Achromobacter xylosoxidans*]	73,00%	7,00E-12	97,00%
***Methanosarcina mazei* 1.H.A.2.6**			
MULTISPECIES: chloramphenicol acetyltransferase CAT [*Enterobacteriaceae*]	100,00%	1,00E-66	100,00%
chloramphenicol acetyltransferase [*Pseudoflavonifractor capillosus*]	100,00%	2,00E-66	99,00%
hypothetical protein [*Escherichia coli*]	100,00%	2,00E-66	99,00%
chloramphenicol acetyltransferase [*Enterobacter hormaechei* subsp. oharae]	100,00%	3,00E-66	99,00%
chloramphenicol acetyltransferase [*Escherichia coli*]	100,00%	3,00E-66	99,00%
chloramphenicol acetyltransferase CAT [*Klebsiella pneumoniae*]	100,00%	4,00E-66	99,00%
Chloramphenicol acetyltransferase (fragment) [*Nitrolancea hollandica* Lb]	100,00%	4,00E-66	99,00%
Group A chloramphenicol acetyltransferase [*Escherichia coli* PCN033]	100,00%	4,00E-66	99,00%
hypothetical protein [*Escherichia coli*]	100,00%	4,00E-66	99,00%
chloramphenicol acetyltransferase [*Enterobacter hormaechei*]	100,00%	4,00E-66	99,00%
integrase [*Methanosarcina mazei*]	82,00%	2,00E-107	99,00%
integrase [*Escherichia coli*]	62,00%	4,00E-79	100,00%
phage integrase family protein [*Escherichia coli*]	62,00%	2,00E-78	100,00%
site-specific integrase [*Escherichia coli*]	62,00%	3,00E-78	100,00%
site-specific integrase [*Escherichia coli*]	62,00%	5,00E-78	100,00%
site-specific integrase [*Escherichia coli*]	62,00%	6,00E-78	99,00%
site-specific integrase [*Escherichia coli*]	62,00%	6,00E-78	99,00%
site-specific integrase [*Escherichia coli*]	62,00%	9,00E-78	99,00%
integrase [*Escherichia coli*]	62,00%	9,00E-78	100,00%
phage integrase family protein [*Escherichia coli* 2875000]	62,00%	9,00E-78	99,00%
hypothetical protein HMPREF9543_03549 [*Escherichia coli* MS 146-1]	100,00%	6,00E-43	100,00%
MULTISPECIES: hypothetical protein [Bacteria][Archaea]	87,00%	5,00E-36	98,00%
hypothetical protein [*Pseudomonas aeruginosa*]	87,00%	4,00E-35	97,00%
hypothetical protein [*Shewanella putrefaciens*]	87,00%	6,00E-35	97,00%
hypothetical protein [*Escherichia coli*]	77,00%	3,00E-31	100,00%
mob-like protein [*Escherichia coli* 541-1]	61,00%	5,00E-22	98,00%
mob-like protein [*Burkholderia cenocepacia*]	61,00%	5,00E-22	98,00%
mob-like protein [*Escherichia coli* 75]	61,00%	6,00E-22	98,00%
hypothetical protein GCWU000182_02011 [*Abiotrophia defectiva* ATCC 49176]	61,00%	6,00E-22	98,00%
conserved hypothetical protein [*Vibrio campbellii* HY01]	61,00%	6,00E-22	98,00%
lac operon repressor [Cloning vector pCALc]	100,00%	0.0	100,00%
lactose operon repressor [*Escherichia coli* HS]	100,00%	0.0	99,00%
lac repressor [*Shigella sonnei*]	100,00%	0.0	99,00%
lactose operon repressor [*Escherichia coli* UMEA 3174-1]	100,00%	0.0	99,00%
lactose operon repressor [*Escherichia coli* DEC6C]	100,00%	0.0	99,00%
lac repressor [*Shigella sonnei*]	100,00%	0.0	99,00%
transcriptional regulator, LacI family [*Escherichia coli* W]	100,00%	0.0	99,00%
lactose operon repressor [*Escherichia coli*]	100,00%	0.0	99,00%
lac repressor [*Shigella sonnei*]	100,00%	0.0	99,00%
lac repressor [*Shigella sonnei*]	100,00%	0.0	99,00%

TABLE V

GC content from archaea genomic compartments

Genome	Genome GC content	Cluster GC content	Contig GC content	diff G-Cluster	diff G-contig
Haloarcula amylolytica JCM 13557	62,13	61,91	61,04	0,22	1,09
Haloarcula argentinensis DSM 12282	61,12	60,59	61,26	0,53	-0,15
Haloarcula californiae ATCC 33799	60,82	61,06	57,72	-0,24	3,1
Haloarculaceae Harcel1	65,74	62,64	65,74	3,1	0
Haloarcula vallismortis ATCC 29715	61,8	54,22	61,86	7,58	-0,07
Halobellus rufus CBA1103	64,12	62,39	58,7	1,73	5,42
Halobiforma haloterrestris DSM 13078	65,43	61,23	56,54	4,21	8,89
Halogranum rubrum CGMCC 1.7738	62,2	60,84	58,94	1,37	3,27
Halogranum salarium B1	62,2	60,76	55,14	1,44	7,07
Halomicrobium zhouii CGMCC 1.10457	66,22	62,5	65,39	3,72	0,84
Halopenitus persicus CBA1233	66,56	63,55	66,56	3,01	0
Halopenitus persicus DC30	65,49	62,29	57,04	3,2	8,45
Haloplanus natans DSM 17983	65,01	60,2	58,63	4,8	6,38
Halorientalis persicus IBRC-M 10043	63,46	64,37	59,01	-0,91	4,45
Halorientalis regularis IBRC-M 10760	65,03	62,87	61,87	2,16	3,15
Halorientalis sp. IM1011	65,58	63,91	65,96	1,68	-0,37
Halorubrum chaoviator DSM 19316	66,48	61,3	57,97	5,18	8,52
Halorubrum ezzemoulense DSM 17463	66,6	60,99	65,5	5,62	1,11
Halorubrum ezzemoulense LD3	66,39	61,12	62,32	5,27	4,07
Halorubrum ezzemoulense LG1	66,07	61,33	62,08	4,73	3,99
Halorubrum sp. BV1	66,01	61,88	58,81	4,13	7,2
Halorubrum sp. SD683	69	61,49	62,21	7,51	6,79
Halorubrum vacuolatum DSM8800	62,9	59,48	55,26	3,41	7,63
Methanobrevibacter ruminantium M1	32,64	34,95	32,64	-2,31	0
Methanobrevibacter sp. RUG344#1	35,15	39,43	35,96	-4,28	-0,82
Methanobrevibacter sp. RUG344#2	35,15	46,55	35,54	-11,4	-0,39
Methanobrevibacter sp. RUG648	32,65	35,82	32,19	-3,17	0,46
Methanobrevibacter sp. RUG833#1	33,62	46,24	33,55	-12,63	0,06
Methanobrevibacter sp. RUG833#2	33,62	32,69	32,27	0,93	1,35
Methanosarcina mazei 1.H.A.2.3	41,61	50,86	51,94	-9,24	-10,33
Methanosarcina mazei 1.H.A.2.6	41,6	50,44	51,44	-8,84	-9,83

Each diff column shows the GC content difference between the whole genome with the tRNA gene cluster region and the contig harboring the tRNA gene cluster, respectively.

TABLE VI

Genomic coordinates of the putative provirus identified

Genome	tRNA cluster region	Proviral region
Haloarcula vallismortis ATCC 29715	254165 - 257733	240536 - 285084
Halorientalis sp. IM101	2239040 - 2244716	2238542 - 2279917
